# Resident-Memory CD8 T Cells and mTOR: Generation, Protection, and Clinical Importance

**DOI:** 10.3389/fimmu.2015.00038

**Published:** 2015-02-05

**Authors:** Ryan T. Sowell, Amanda L. Marzo

**Affiliations:** ^1^Department of Immunology and Microbiology, Rush University Medical Center, Chicago, IL, USA

**Keywords:** memory, CD8 T cells, tissue resident, mTOR, mucosa

Tissues such as the lung, skin, intestinal epithelium, and reproductive tract serve as a barricade against pathogen exposure for the entire body. Specifically within the skin and mucosal tissues, a population of resident CD8 T cells plays a salient role in the protection against infection. Resident-memory CD8 T cells (T_RM_) are a long-lived subset of memory CD8 T cells that do not re-circulate after taking up residence in the tissues. Traditionally memory CD8 T cells were conceptually divided into two subsets central (T_CM_) and effector (T_EM_) memory where T_CM_ preferentially localized within secondary lymphoid tissues (SLO) and T_EM_ circulated throughout the peripheral tissues. While the concept of T_CM_ and T_EM_ has been considerably explored, memory CD8 T cells found within barrier tissues do not totally fit within the T_CM_/T_EM_ paradigm. Through the study of circulating CD8 T cells, our understanding of memory CD8 T cells has grown tremendously in the last 25 years. We now understand that it is not sufficient to simply generate large numbers of circulating memory CD8 T cells in order to enhance protection against localized infection. Developing clinical strategies that can enhance protection against mucosal pathogens will require a clear understanding of how memory CD8 T cells are generated and maintained within barrier tissues at the sites of initial pathogen exposure. We will outline our current understanding of T_RM_ in respect to their generation, functional importance, and how future studies must shed light on how we can exploit T_RM_ to develop the next generation of effective vaccines.

## Generation of Tissue-Resident- Memory CD8 T Cells

CD8 T cells are primed within tissue draining lymph nodes and lymphoid tissues. Once primed, CD8 T cells receive tissue-specific signals that allow them entry into tissues, which at steady-state are normally non-permissive to T cell migration (1). Upon entry into mucosal or skin tissues CD8 T cells take up residence and do not re-circulate (2, 3). For T cells to enter the small intestine the integrin α4β7 and chemokine receptor CCR9 are important (4, 5). α4β7 and CCR9 expression is induced by dendritic cell-derived signals like the vitamin A metabolite retinoic acid (6). α4β7 is transiently expressed and the timing of its expression correlates with the window of opportunity for T cell migration into the small intestine (2, 4, 5). Primed T cells that enter mucosal tissues differentiate into T_RM_ in response to tissue-derived signals, not limited to but including TGF-β and IL-15 (5, 7). Tissue-derived signals like TGF-β and IL-15 are not uniquely confined to barrier tissues, as their availability is also important for circulating CD8 T cells. T_RM_ originate from KLRG1^−^ effector cells, are not terminally differentiated, however, express lower levels of CD127 and CD122 than circulating memory CD8 T cells (7, 8). While T_RM_ share a common naïve precursor with blood and SLO memory cells they are inherently different from their circulating counterparts (9). Unlike circulating memory CD8 T cells, T_RM_ maintain expression of CD69 and elevated levels of Granzyme B, attributes akin to effector cells (2, 10, 11). Generation of T_RM_ is dependent on CD69 as overexpression *S1pr1* or deletion of CD69 in CD8 T cells limits T_RM_ formation (7, 12). T_RM_ also upregulate the integrin subunit CD103 whereas circulating memory CD8 T cells remain CD103^−^ (2, 8). A qualitative feature that distinguishes circulating T_CM_ and T_EM_ from T_RM_ is that regardless of infection or anatomical location T_RM_ share a signature of core gene transcripts (7). Within this signature are genes involved in chemotaxis, adhesion, and co-stimulation. Expression of some genes associated with circulating memory cells are decreased, e.g., *Eomes, S1pr1*, and *Ly6C*. The signaling events that regulate the transcriptional programing of T_RM_ is unknown.

## mTOR and T_RM_ Formation

The mammalian target of rapamycin (mTOR) kinase is a central regulator of many cell processes, including survival, differentiation, and proliferation. It orchestrates cellular responses to external and internal environmental cues. Signaling through the mTOR pathway is an important event for memory formation (13). During each stage of T cell priming, mTOR is activated i.e., TCR/CD28/IL-12. However, inhibiting mTOR in effector CD8 T cells increases the number of memory CD8 T cells in the circulation by increasing the number of effector CD8 T cells that are CD127^hi^KLRG1^−^. This suggests that while mTOR is needed early during activation for effector formation, at some point during the effector phase mTOR could be attenuated or turned off in order for effector cells to progress toward memory differentiation.

We recently reported that accumulation of CD8 T cells in the small intestine and FRT is critically dependent on prolonged mTOR signaling (14). We showed that blocking mTOR signaling during the effector phase inhibits the accumulation of effector CD8 T cells in the mucosa thereby limiting T_RM_ formation. This suggests that mTOR controls signals that either direct the migration of CD8 T cells into these barrier tissues or influences their survival. More importantly, because one naive CD8 T cell can give rise to memory in both the SLO and mucosal, our observations indicate that mTOR may regulate the divergence between circulating and resident-memory CD8 T cells. It is also possible that mTOR controls the context in which memory precursors receive signals from cytokines and growth factors. Circulating memory CD8 T cells interact with different stromal and hematopoietic cells than tissue-resident-memory CD8 T cells and this could influence their overall function and survival. For example, IL-15 is an important homeostatic cytokine for memory CD8 T cells, received through a contact-dependent mechanism known as trans-presentation, and for T_RM_ formation (5, 15). Within the memory CD8 T cell populations both IL-15-dependent and IL-15 independent populations have been described. mTOR can be activated by IL-15 and inhibition of mTOR leads to a predominantly IL-15 independent memory population (16, 17).

Many cell types within different tissues produce IL-15 and as a result T_RM_ may receive IL-15 signals more frequently than circulating T_EM_ that may only receive transient IL-15 signals when they circulate through the tissues. While TGF-β induces apoptosis in circulating effector CD8 T cells, T_RM_ generated from effector CD8 T cells rely on TGF-β signals (7, 18). Since mTOR has broad affects on cell physiology and is activated in effector CD8 T cells, mTOR may be central in regulating the unique transcriptional program in T_RM_. mTOR signaling is mediated by two distinct complexes, mTORC1 and mTORC2 that control responses to environmental cytokine milieus (13). In the absence of mTOR, T cells are unable to respond to cytokines that direct their differentiation (19). The differentiation of naïve CD4 T cells into Th1 and Th2 subsets, requires mTORC1 and mTORC2 signaling, respectively. Loss of mTORC1 signaling in CD4 T cells blocks their ability to upregulate T-bet in the presence of IL-12 (20). IL-12 mediated mTORC1 signaling in CD8 T cells sustains the expression of T-bet and promotes differentiation into effector cells (21). Moreover, inhibiting mTORC1 increases the expression of Eomes and skews memory CD8 T cells toward IL-15 dependence (17). CD4 T cells that lack mTORC2 signaling have diminished responses to IL-4 and IL-13. Less is known about the function of mTORC2 in CD8 T cells. However, given CD4 and CD8 T cells’ shared dependence on the same cytokines or cytokine signaling pathways for their differentiation, the role of mTORC2 in CD8 T cell function will be an important direction for future study.

## Are T_RM_ Required for Protection?

A major question is whether T_RM_ positioned at sites of exposure are critical to protect against local re-exposure. The presence of T_RM_ can provide local protection (3). However, circulating memory CD8 T cells can migrate into barrier tissues upon re-challenge and provide protection. However, antigen must first make its way to mucosal draining lymph nodes in order to prime circulating memory CD8 T cells and recruit them to infected tissues. Due to their proximity to infected tissue T_RM_ may be able to respond a pathogen exposure more rapidly than circulating memory CD8 T cells (22). The mucosa is the major route of entry for HIV infection. Within a few hours to days after exposure, HIV can breach the mucosal barrier, infect resting CD4 T cells, and presumably establish latent virus reservoirs (23). In non-human primate models of infection, the latent reservoir is seeded rapidly sometime during the first 3 days of infection creating a big challenge for the immune system (24). The time required for circulating memory CD8 T cells to mount a response may not be quick enough to prevent the establishment of HIV infection. It is now evident that eliminating infected founder cell populations in the area of initial HIV entry is a critical requirement for the immune system to provide protection. Robust T_RM_ responses at the portal of HIV entry may be critical component. Whether T_RM_ can directly kill local infected cells is not explicitly known. The surface area of the mucosa barrier is immense and it is estimated that for every T_RM_ cell there could be tasked to survey as many as ~10^3^ cells (25). Therefore, it is important to know the concentration of T_RM_ that would constitute a critical mass to provide protection. However, recent work has shown that T_RM_ may do more than kill infected cells. T_RM_ can rapidly respond to local challenge and recruit new antigen-specific memory CD8 T cells from the circulation as well as bystander cell populations (26). Moreover, T_RM_ can activate neighboring NK cells and B cells by providing a local source of IL-2, IFN-γ, and TNF-α (27). Collectively, these recent findings suggest that T_RM_ could provide protection not only by killing of infected cells in barrier tissues, but also by taking part in pathogen sensing and initiation of the immune response. Thus it is important to know whether vaccine prime-boost regimens, which can generate large numbers of T_RM_, will ultimately lead to protection against mucosa-acquired infections such as HIV and HSV.

## Manipulating T_RM_ for Clinical Benefit

The observation that upon reactivation T_RM_ are capable of inducing activation of innate cells and even protecting against an antigenically unrelated pathogen may have broader implications for vaccine design (27). It is understood that local priming of CD8 T cells or priming within mucosa draining lymph nodes can generate effector CD8 T cells that can become T_RM_. Building vaccines that favor priming at these tissue sites and the establishment of long-lived T_RM_, while challenging, is important, and may include the following strategies. Orally administered vaccines can induce CD8 T cells that accumulate within tissues such as the female reproductive tract and small intestine of the gastrointestinal tract, while attractive the longevity and functional attributes of the memory cells generated from this approach need to be further elucidated (28). Prime-boost strategies using local challenge potentially can increase the number of T_RM_ to a level that can provide protection. Identifying key regulators of T_RM_ formation, as candidate adjuvants to more traditional vaccine strategies, in order to re-direct primed effectors to mucosal tissues to generate increased numbers of T_RM_. However, the ability of these re-positioned T_RM_ to persist like their counterparts that are generated *in situ* is still an area of research that warrants further investigation. On the other hand, CD8 T_RM_ cells may also play a central role in tissue destruction in organ-specific autoimmune disorders. The same factors that are important for the generation of T_RM_ may be potential targets for blocking pathogenic CD8 T cell responses in the small intestine of patients with celiac disease. Our findings that low-doses of rapamycin, a pharmacologic mTOR inhibitor, blocked the accumulation of CD8 T cells within the intestinal mucosa suggest the mTOR pathway as a candidate for therapeutic intervention (14). Moreover, we used a model of T cell mediated enterocyte destruction that may have some similarities to celiac disease pathogenesis. Using this model, we demonstrated that rapamycin was capable of blocking the accumulation of CD8 T cells specific for antigen expressed exclusively within the small intestine. In this model, the autoimmunity becomes fatal upon addition of systemic inflammation and the administration of low-dose rapamycin reversed intestinal destruction and enhanced survival. For tumor immunity one hurdle is positioning enough functional CD8 T cells within the tumor milieu. Interestingly, a small population of memory CD8 T cells has been identified in the spleen and lymph nodes that share characteristics of T_RM_ in that they do not re-circulate, and express CD69 (29). It is of significant interest to determine if an equivalent population of T_RM_ populates the tumor and if so how to manipulate these cells to the point where they have a negative impact on tumor growth.

## Conflict of Interest Statement

The Associate Editor, Kimberly Sue Schluns, declares that despite having collaborated with author Ryan T. Sowell in the past 2 years, there has been no conflict of interest during the handling of this manuscript. The authors declare that the research was conducted in the absence of any commercial or financial relationships that could be construed as a potential conflict of interest.
